# A Risk Assessment of Aflatoxin M1 Exposure in Low and Mid-Income Dairy Consumers in Kenya

**DOI:** 10.3390/toxins10090348

**Published:** 2018-08-29

**Authors:** Sara Ahlberg, Delia Grace, Gideon Kiarie, Yumi Kirino, Johanna Lindahl

**Affiliations:** 1 Department of Biosciences, International Livestock Research Institute, P.O. Box 30709, Nairobi 00100, Kenya; sarahellinahlberg@gmail.com (S.A.); d.grace@cgiar.org (D.G.); 2 Department of Food and Environmental Sciences, University of Helsinki, P.O. Box 66, FI-00014 Helsinki, Finland; 3 Mount Kenya University, P.O. Box 342, 01000 Thika, Kenya; gmwangi061@gmail.com; 4 Department of Veterinary Sciences, University of Miyazaki, 1-1 Gakuen Kibanadai-nishi, Miyazaki 889-2192, Japan; kirinoyumi@cc.miyazaki-u.ac.jp; 5 Department of Medical Biochemistry and Microbiology, Uppsala University, Box 582, 75123 Uppsala, Sweden; 6 Department of Clinical Sciences, Swedish University of Agricultural Sciences, P.O. Box 7054, 75007 Uppsala, Sweden

**Keywords:** urban consumers, cancer, stunting, milk, dairy products

## Abstract

Aflatoxin M_1_ (AFM_1_), a human carcinogen, is found in milk products and may have potentially severe health impacts on milk consumers. We assessed the risk of cancer and stunting as a result of AFM_1_ consumption in Nairobi, Kenya, using worst case assumptions of toxicity and data from previous studies. Almost all (99.5%) milk was contaminated with AFM_1_. Cancer risk caused by AFM_1_ was lower among consumers purchasing from formal markets (0.003 cases per 100,000) than for low-income consumers (0.006 cases per 100,000) purchasing from informal markets. Overall cancer risk (0.004 cases per 100,000) from AFM_1_ alone was low. Stunting is multifactorial, but assuming only AFM_1_ consumption was the determinant, consumption of milk contaminated with AFM_1_ levels found in this study could contribute to 2.1% of children below three years in middle-income families, and 2.4% in low-income families, being stunted. Overall, 2.7% of children could hypothetically be stunted due to AFM_1_ exposure from milk. Based on our results AFM_1_ levels found in milk could contribute to an average of −0.340 height for age z-score reduction in growth. The exposure to AFM_1_ from milk is 46 ng/day on average, but children bear higher exposure of 3.5 ng/kg bodyweight (bw)/day compared to adults, at 0.8 ng/kg bw/day. Our paper shows that concern over aflatoxins in milk in Nairobi is disproportionate if only risk of cancer is considered, but that the effect on stunting children might be much more significant from a public health perspective; however, there is still insufficient data on the health effects of AFM_1_.

## 1. Introduction

Contaminants in foods causing health problems include pathogens and toxins, which are present in raw materials or introduced during processing. Aflatoxins are mycotoxins produced by certain fungi, ubiquitous in soils in tropical and sub-tropical areas. The maximum level for aflatoxins in foods are regulated in many countries due to their harmful effects on health, though the allowable limits vary [1]. Aflatoxins, including aflatoxin B_1_ (AFB_1_) and aflatoxin M_1_ (AFM_1_), are the most potent carcinogens among all mycotoxins and are classified as Group 1, meaning they have been proven to be carcinogenic to humans [2].

The European Union (EU) regulation 1881/2006 [3] set the legal maximum limit for AFM_1_ in raw milk at 0.05 ng/g, which is lower by one order of magnitude than the Codex Alimentarius recommendation [4] of 0.5 ng/g. The Codex recommendation is assumed to be followed in the Kenyan standards, although there is some confusion among stakeholders as to which aflatoxin standard applies to milk [5].

In uncontrolled and unmonitored food production and distribution systems, aflatoxin levels in foods can rise to alarming levels, resulting in acute and sometimes fatal illness. Aflatoxin B_1_ prevalence is variable and affected by season, weather, geographic area, and storage conditions, among other factors [6]. AFM_1_ is the 4-hydroxy derivative of AFB_1_, and the major toxin metabolite found in milk and urine in animals and humans exposed to dietary AFB_1_ [7]. AFM_1_ is considered at least 10 times less carcinogenic than AFB_1_, based on animal trials [8,9].

Severe aflatoxin poisoning, called acute aflatoxicosis, caused by consumption of large amounts of aflatoxins, has occurred several times in Kenya resulting in hundreds of fatalities [6,10,11]. These cases have increased awareness of the prevalence of aflatoxin in the feed and food chains leading to policy change, public concern, research efforts, and mitigation interventions.

Carcinogenic effects have mainly been studied for AFB_1_, but all aflatoxins are believed to be carcinogenic [2]. Aflatoxins are associated with liver cancer, which was estimated to have caused 745,000 deaths in 2012, mostly due to hepatocellular carcinoma (HCC) [12]. Similarly, Wong et al. [13] estimated a global total of 782,451 new liver cancer cases and 745,533 related deaths per year based on cancer reporting in 2012. Less developed regions bear 95% of the total liver cancer incidences and 96% of the mortality [13]. Risk factors for HCC include being male, lower socioeconomic status, and poverty [13]. Infection with the hepatitis B virus (HBV) is one of most important risk factors. In hepatitis B negative (HBsAg-negative) and hepatitis B positive (HBsAg-positive) populations, the burden of HCC cases attributable to aflatoxins exposure worldwide, through maize and peanuts consumption, was estimated to be 11–450 and 44–2270 annually, respectively [14]. Gibb et al. [15] estimated 22,000 (95% UI 9000–57,000) aflatoxin-related HCC cases globally in 2010 using the population attributable fraction approach. Another approach found aflatoxin-attributable liver cancer burden globally to be 25,500–155,000 cases annually [16]. In the African region, it was estimated that aflatoxins cause 0.4 (0.1–1) deaths per 100,000 people annually [15].

Stunting, based on low height-for-age z-score (HAZ), is defined when height is more than two standard deviations (SD) below the standard mean [17]. The HAZ score is a metric showing how many standard deviations a child is from the mean height-for-age, and a HAZ of −2 means that a child is stunted (more than two standard deviations below mean height); a HAZ of −3 is considered severe stunting. Stunting is a well-established risk marker of poor child development and indicates chronic malnutrition; it has been associated with chronic aflatoxin exposure [18,19]. Stunting and growth impairment are major concerns [19,20] as stunting has serious impacts beyond childhood resulting in lower school achievements, life-time earnings, increased health problems, and decreased productivity [18,21]. Aflatoxin exposure, due to the suppression of the immune system causing increased risk of infections or due to direct effects in the gut or liver, could potentially cause or accelerate stunting risk and severity [18].

The AFB_1_ exposure association with stunting is considered likely to be causal, but the mechanisms are yet to be proven and there are studies indicating a negative association between AFB_1_ exposure and growth impairment or stunting [19,22,23,24,25] as well as studies where association between AFB_1_ exposure and growth rate was not observed [26,27,28]. The variety in exposure levels and reduced growth levels suggest a possible threshold of aflatoxins for observable growth impairment effect. However, it should be noted that despite the association between aflatoxin exposure and growth impairment, many other factors have an influence on undernutrition, child development, and toxicity effects, including health status, nutritional intake, food quality, poor sanitation, and general poverty [19,22,25,29,30].

In previous studies, AFM_1_ exposure in early life and childhood was associated with reduced HAZ score in children [27], reduced birth weight [31], reduced height at birth [32], and stunted growth [33]. However, in the case of AFM_1,_ there are fewer studies on the association with growth and no proven causality or mechanism between stunting and exposure, which means that any risk assessment for stunting is purely hypothetical.

World Health Organization (WHO) estimations of the global and regional disease burden of foodborne chemical toxins [15] consider two approaches, top-down and bottom-up, for assessing aflatoxin health burden and discuss why these differ. The top-down approach is based on estimations of actual death and mortality cases, whereas the bottom-up approach uses exposure levels of diets and contamination levels in foods to predict death and mortality [15]. Both approaches are prone to biases: in particular, regional cancer registration data likely under-estimate cases due to limited health care and failure of cancer diagnosis or under-reporting, especially in less-developed regions, whereas predictive approaches may over-estimate cases [13].

Risk assessment of a chemical or compound through dietary exposure includes hazard identification, hazard characterization, exposure assessment, and risk characterization [34]. In this study, we conducted a predictive (bottom-up) risk assessment for AFM_1_ exposure, stunting, and cancer risk among urban milk consumers in Nairobi, Kenya. Dietary exposure was derived from studies conducted during 2013–2016 in Nairobi, Kenya, analyzing AFM_1_ levels in formal and informal dairy products, milk consumption levels, and exposure of adults and children. Stunting risk was assessed based on exposure and previous stunting prevalence [27]. To assess the risk of cancer caused by dietary exposure to AFM_1_ through consumption of milk and milk products, exposure levels were calculated, and data on estimated cancer cases were used.

## 2. Results

### 2.1. Milk Consumption of Adults

The analysis of milk consumption shows differences between consumer groups based on their income status varying from 148 L annually in mid-income areas up to 240 L annually in low-income areas. Table 1 shows the average consumption of milk by adults in low- and mid-income areas based on self-assessments, portion estimations, and 24-h dietary recalls. From mid-income adult respondents, 44% reported no milk consumption compared to 18% in low-income respondents. Similarly, respondents in mid-income areas reported lower daily milk intake than in low-income areas, 229 mL/day and 539 mL/day on average among all respondents, respectively.

### 2.2. Milk Consumption of Children

Milk consumption for children below 3 years in low- and mid-income areas was calculated combining several surveys using 24-h recall and self-reporting. The milk type was not specified in the studies focusing on milk consumption of children. Table 2 shows the average reported milk consumption among children in low- and mid-income households. The average values show differences in consumption between areas.

### 2.3. AFM_1_ Levels in Raw and Processed Milk Samples

Table 3 summarizes the combined data of all AFM_1_ analyses (N = 619) from the studies and mean levels of AFM_1_ levels for different product groups collected from different income areas. Only 19 samples had levels above 0.5 ng/g of AFM_1_. Only three samples (3/619) were not contaminated with detectable AFM_1_, and 99.5% were positive for aflatoxins, with the contamination level ranging from 0 to 2.55 ng/g. The median for the AFM_1_ levels was lower than the mean, reflecting the large standard deviation (SD), so the few samples with very high concentration raised the mean.

### 2.4. Exposure Assessment of Adults

Exposure to AFM_1_ from milk consumption was assessed based on milk consumption averages in different income groups and average of AFM_1_ levels in milk and milk products. In Table 4, the exposure levels of adults are summarized, using the mean contamination levels (Table 3) and the mean consumption levels (Table 1).

### 2.5. Exposure Assessment of Children

Exposure assessment for AFM_1_ from milk products was calculated for children below three years old (Table 5) using the mean contamination levels (Table 3) and the mean consumption levels (Table 2). The exposure was calculated based on milk consumption in different income areas and in AFM_1_ levels found in milk and milk products.

### 2.6. Assessment of Cancer Risk

For cancer risk assessment, estimations are summarized in Table 6 of AFM_1_-induced cancer risk in different socioeconomic consumer groups exposed to AFM_1_ in milk. The Kenyan population is estimated to be 46 million [35].

### 2.7. Risk Assessment of Stunting

The growth reduction estimation for children below three years exposed to AFM_1_ from milk, based on different consumption levels of milk in different income areas and AFM_1_ levels in milk is summarized in Table 7. In average, AFM_1_ can have an effect of −0.340 on height-for-age z-score, contributing to 2.7% of childhood stunting (−2 or more reduction in height-for-age z-score).

## 3. Discussion

This risk assessment used milk consumption and milk contamination data from several studies conducted in Nairobi in order to understand the potential impact of aflatoxin contamination on the health of the urban population. While this analysis included observations from several surveys, the assessment is not as strong as it could have been if it were possible to include the same number of participants and directly measure milk consumption and test the different products consumed directly. This approach would have allowed confidence ranges using deterministic exposure assessments. Despite this, the levels used for the risk assessment reflect the distribution of samples in Nairobi, and the reported consumption is from consumers purchasing milk in the same area.

### 3.1. Milk Consumption

Based on our results, daily average milk consumption was estimated to be approximately 440 mL in adults, with low-income milk consumers consuming more (660 mL) than mid-income consumers (400 mL). The estimate of milk consumption in low-income areas may have been biased because some of the interviewed were milk traders, who have better access to milk. However, the significant number of mid-income participants stating no milk consumption (44%) is in line with lower averages in consumption levels. The decreasing consumption of liquid milk and replacement of traditional foods with high-value (processed) products along with increasing income is a global phenomenon.

Contradicting the milk consumption of adults, mid-income children below three years old consumed more milk daily (630 mL) than low-income children (400 mL). The observed variance among low-income children is higher than the average indicating wide disparity among milk intake in low-income areas. This is consistent with a common belief that milk is especially suited to children.

However, this study was not concerned with the origin of the milk, but merely draws attention to the potential risk effects of aflatoxins associated with milk consumption on urban consumers. The confidence intervals of the estimates overlap, so a difference cannot be definitively claimed. The different methods used to obtain the consumption data (24 h recall and self-reporting) produced different estimates, with the studies using self-reporting estimating the consumption higher than studies using 24 h recall (complementary data). These differences were also to be expected.

### 3.2. AFM_1_ Levels

The prevalence and levels of AFM_1_ in milk and milk products in urban Nairobi are concerning. Aflatoxin levels in different product groups available in different income areas showed a trend of lower aflatoxin levels in products available in mid-income area and in all processed milk samples. The lower aflatoxin levels in processed milk samples could be the result of formal monitoring and control systems, although we do not have evidence of the extent to which these are practiced in Kenya. Clearly, the lack of any monitoring systems in informal markets enables contaminated products to be available in the markets.

Whether the lower aflatoxin levels in processed and mid-income area samples are due to stricter control or different production systems, there are still challenges. Only 3% of the samples were non-compliant with detected concentrations above the limit of 0.5 ng/g AFM_1_ in milk, but 56% of the samples had AFM_1_ concentrations above 0.05 ng/g. All mean levels in all categories were above 0.05 ng/g. Although processed milk samples had with lower AFM_1_ levels, 49% were above 0.05 ng/g. It is not clear which level Kenya officially follows, which is creating confusion among stakeholders in the markets.

Exposure to AFM_1_ is likely a long-standing problem, and during past 10 years, no improvement has been observed in the contamination prevalence, with almost all milk being contaminated with AFM_1_ [10,36,37,38,39,40].

In the global context, AFM_1_ levels found in Kenyan milk are high. Milk in Europe is most often analyzed for AFM_1_, but is also the safest. The least amount of data is available from African countries, but the available data imply highest prevalence and frequent detection levels [41,42]. In Brazil, 83% of the milk samples tested positive for AFM_1_, in a range of 0.008 to 0.760 ng/g [43] and in India, almost half of the analyzed milk was contaminated, with 44% being above EU limit [44].

### 3.3. Exposure

Total estimation of AFM_1_ exposure was 46 ng/day on average (0.8 ng/kg bw/day). Low-income consumers had higher estimated exposure levels, at 69 ng/day (1.2 ng/kg bw/day), than the mid-income consumers at 43 ng/day (0.7 ng/kg bw/day). The difference in exposure levels can be explained by lower milk intake levels among mid-income consumers, and lower levels of AFM_1_ analyzed in samples acquired from middle income areas. Sources of potential inaccuracy in these estimates include: milk consumption reported by respondents could be inaccurate, consumption data focused only on liquid milk, consumers of one income bracket may purchase milk in areas of another income bracket, AFM_1_ content most likely varies between batches, and there may be seasonal differences [45]. However, overall exposure to AFM_1_ from milk seems to be high and chronic.

Calculated exposure levels of children below three years to AFM_1_ were significantly higher than in adults, with the same total intake (46 g/day) but higher intake per bodyweight (3.5 ng/kg bw/day), due to relatively high average milk consumption and low body weight. Adults and children in low-income areas were more exposed to AFM_1_, especially when consuming milk sourced from low-income areas. Mid-income children were estimated to consume 41 ng/day (3.2 ng/kg bw/day) of AFM_1_ through milk sourced from mid-income areas compared to the exposure of 47 ng/day (3.6 ng/kg bw/day) in low-income children consuming milk sourced from low-income areas.

Another study of milk consumption and AFM_1_ concentration in the milk samples [36] estimated the daily exposure to AFM_1_ from milk at 94 ng/per day for children and 120 ng/day for adults, which is even higher than our estimations, but the study focused on milk retailers’ households where the milk consumption was reported to be significantly higher (900 mL/day for adults and 730 mL/day for children).

The Codex Alimentarius committee compared the consequences of setting the maximum allowable limit to 0.05 ng/g versus 0.5 ng/g for AFM_1_ in milk. The recommended standard 0.5 ng/g was based on the data available summarizing the estimated exposure levels; intakes of AFM_1_ from milk was estimated 0.030 ng/kg bw/day and based on milk consumption levels exposure was estimated to be 0.023 ng/kg ng/kg bw/day when a maximum level of 0.5 ng/g was used, and 0.0035 ng/kg bw/day for a maximum level of 0.05 ng/g [46]. Clearly, the exposure levels in urban Nairobi are significantly higher.

### 3.4. Cancer Risk

The results show a low risk for cancer due to AFM_1_ exposure from milk consumption for adults. Assuming levels and consumption were similar throughout Nairobi (a reasonable assumption), there would be 0.04 cases per year for an urban population of 1,000,000 (26% of total population [35]), which would translate to less than two cases per year for the whole of Kenya, assuming the exposure was similar throughout the population, which is unlikely. The estimates are, however, more uncertain than those for AFB_1_, since there is more uncertainty about the carcinogenicity of AFM_1_. In this study, we assumed that the potency was 10 times lower, which is based on data from rodent trials [8].

Even though the cancer risk from AFM_1_ was low in this study, the effects of AFM_1_ on health, and especially the combined effects of mixtures of mycotoxins, aflatoxins, other dietary contaminants, alcohol consumption, and poor diet on cancer risk still remains largely unknown. The combined exposure to different aflatoxins, mycotoxins, and other contaminants in foods might cause more significant or unknown risks [15]. There is a possibility of a cumulative effect. Still, there does seem to be a disconnection between the levels of expressed concern of consumers over aflatoxin in milk [47] and the relatively low estimated mortality. Consumers often appear to have higher concern over chemicals in food, although experts generally agree that biological hazards present greater risk [48].

### 3.5. Growth Reduction

Based on our findings, levels of AFM_1_ exposure from milk could contribute to HAZ reduction of −2 or more in 2.7% of children. The mean average growth reduction in HAZ score from AFM_1_ exposure from milk would be −0.340. Mahdavi et al. [33] reported a −0.31 HAZ z-score reduction in infants below three months consuming breastmilk with an AFM_1_ mean concentration of 9.69 pg/mL, which is in line with our findings. Aflatoxin M_1_ exposure was reported to be inversely related to growth in infants below six months, with a −0.013 z-score reduction in HAZ with increasing exposure [49]. This study found a higher exposure (11.3 ng/kg bw/day) than we observed, but our observation resulted in a more significant reduction in height-for-age z-score among older children (up to three years). Abdulrazzaq et al. [50] found a strong negative correlation between AFM_1_ levels both in umbilical cord blood and maternal serum and birth weight of the infants. Again, AFM_1_ was detected in 98% of samples with a median concentration of 8.2 ng/kg in breastmilk (*n* = 160), and was associated inversely with height of infants at birth [32].

All these studies focused on infants and breastmilk, whereas ours focused on children consuming bovine milk. Moreover, although several studies showed associations between aflatoxin and stunting, correlation does not imply causation, and it is still not definitively proven that aflatoxin contributes causally to stunting, or the magnitude, if any, on the effect on growth. In addition, estimates of contribution to stunting or based only on the effects of AFM_1_, not considering that increased milk consumption by itself promotes child growth [51], nor any other dietary, health, or sanitary factors. It is suggested that a daily consumption of 245 mL milk most likely has an additional effect of increasing height by 0.4 cm annually [52]. As observed in previous studies with AFB_1_ and stunting association, varying results from different studies can be due to, among other reasons, the general initial health status of the studied cohort [25,26].

### 3.6. Overall

Risk assessments inevitably simplify complex processes. A number of studies have examined associations between AFB_1_ exposure and stunting, with variable results, but there has been less research on AFM_1_ exposure from milk in young children. Although some studies have analyzed the association between AFM_1_ in breast milk and maternal blood and stunting [32,33,49,50], only one study provided an estimate based on consumption of cow milk. This estimate was used in our study, but the limited number of studies makes the estimate more uncertain [27].

Assuming that the estimate would be correct, and without taking the growth promotion from milk itself into account, our results indicate that aflatoxins could contribute to a non-negligible proportion of stunting cases and severity. Our study did not take any other dietary exposure or health status into consideration. Our results would imply that, when considering aflatoxins in milk, stunting and exposure to AFM_1_ may be a more serious public health consequence than liver cancer, but there is too little evidence to be certain of this. Whether the AFM_1_ can be linked to stunting or not, the exposure levels are evidently high among urban Nairobi children and adults consuming milk, which can be a cause of concern for consumers and policymakers, although not to an extent to deter people from consuming milk.

It is also important to understand the results in the context of the increasing trend in global food trade as no market remains in isolation. Food is traded more than ever [53] and as markets for higher quality food emerge, there is an increasing possibility that poor quality food is channeled to consumers with low purchasing power. Food safety should be a default to all consumers and not be based on socioeconomic status.

However, food security is still an issue in Kenya, and there is a trade-off when applying strict regulatory limits [5]. Optimally, when deciding on the limits to apply in a country, it is recommended that a Margin of Exposure approach be used [54], but in many countries, particularly in low- and middle-income countries, regulatory limits are often adopted from trade partners or driven by public concerns, even when there are few means of implementation. Difficulties in obtaining the current valid standards for food products, including milk, and confusion over the standards for aflatoxin in milk in Kenya is not facilitating implementation. Available and official documentation refer to different levels [5,55,56], which can create frustration, confusion, and ignorance among producers. The costs of purchasing official standards may deter small-scale producers from acquiring them and hence impede implementation. There is an urgent need to have a clear communication about the regulations for the successful control and monitoring implementation among all stakeholders.

Overall, there seems to be no change in the AFM_1_ situation in Kenyan dairy markets since the aflatoxin problem became evident to large community in 2004, directly reflecting the dysfunctional control systems and failed interventions. To strengthen national, safe, and high-quality dairy production now and in the future, drastic changes must happen in the dairy markets.

## 4. Conclusions

We conclude that evidence of the harmful effects of AFM_1_ is scarce, and that more information should be collected in order to warrant the strict standards imposed in many parts of the world. This study also shows that consumers purchasing dairy products from informal markets are more likely to be exposed to AFM_1_ than middle-income consumers purchasing processed products. The focus of future studies should be on exposure from complete diets and a range of contaminants. Also, the economic costs and benefits of standards, and the feasibility of implementation, should be taken into consideration, especially for less developed countries where less strict limits might be in place. Overall, in light of the present evidence on the negative health effects of AFM_1_, this study indicates that milk may contribute to a non-negligible health burden, but that further research should focus on possible impacts on stunting, as this is by far the greatest potential negative health impact.

We acknowledge the limitations and uncertainty within this study. Most important were the limitation of available data and lack of known confounders and mechanisms of how AFM_1_ might cause stunting, either directly or indirectly. Longitudinal, cross-sectional, or ideally a clinical trial and multidisciplinary studies would be required to better understand the effects of AFM_1_ of milk on child development. Even more importantly, measures to generally improve food safety and mitigate food safety hazard prevalence in food and feed chains should be a high priority especially in countries where the burden of foodborne disease is very high.

## 5. Materials and Methods

We conducted a risk assessment for AFM_1_ in milk by combining AFM_1_ exposure data from several studies conducted in low- and mid-income areas in Nairobi County between 2013 and 2016 [27,36,45]. The low-income areas where data were collected were Korogocho and Dagoretti—two informal settlements dominated by informal supply chains. The mid-high-income area of study, Westlands, is characterized by supermarkets and shopping centers and considered an expensive area to live. Income status of the study areas was determined by the reported income of the households: low-income households were those earning less than 20,000 Kenyan Shillings (KES)/month [57] and mid-income areas were identified based on local expert opinion and consensus.

In brief, the different data sets that were summarized included:

(1) Data from a survey among informal milk traders in the low-income area of Dagoretti, Nairobi, which included consumption data of milk-trading families and AFM_1_ levels in raw milk [36]. In total, 200 samples of raw milk were analyzed for AFM_1_ and 250 traders provided data on milk consumption in their families. The milk consumption estimations were self-reported by the families. This study also concluded that most traders supplied milk directly from farms, which means that the source of the milk is close to the trading point. Daily AFM_1_ exposures were calculated.
(2) Data from a survey on milk consumption in children (below 3 years) from two low-income areas in Nairobi (Korogocho and Dagoretti) and the levels of AFM_1_ in the milk they consumed [27,57]. This study contained data on milk consumption for 204 children, of which 41% were stunted, and 128 raw milk samples were analyzed for AFM_1_ with ELISA.
(3) Data on milk consumption in adults and children (below 2 years) in the low-income area of Dagoretti and the mid-high-income area of Westlands [47,58]. In the two areas, 323 and 299 adults, respectively, were interviewed for theirs and their family’s milk consumption habits; results were reported self-estimations.
(4) Data on AFM_1_ levels from milk sampled from raw and processed milk sampled in the low-income area of Dagoretti and the mid-high-income area of Westlands [45]. This study analyzed the levels of AFM_1_ in 291 different milk products, including both raw and processed samples.

Milk consumption estimations were conducted from a 24-h dietary recall study, portion estimations [27,57], and self-reported consumption by the respondents [36,47,58]. For exposure, we used an overall daily milk consumption levels for adults of 437 mL/day, and 657 mL/day for low-income milk consumers and 406 mL/day for mid-income milk consumers. Milk consumption estimates of 438 mL/day for children overall, and 398 mL/day for low-income area children and 626 mL/day for mid-income area children were used.

Exposure was calculated for all the samples, product categories, both income area sources, and respective income area for the consumer group to highlight the differences in exposure. Processed products were all milk products, except raw milk samples, and were also sub-divided between the heat-treated and fermented products. Milk samples from mid-income areas were only processed milk samples, and samples collected from low-income areas included both raw and processed milk.

The exposure was calculated deterministically by multiplying mean contamination level with mean consumption level and divided by body weight of estimated average 60 kg for adults based on mycotoxin safety evaluation for intake [59] and 13 kg for children below 3 years old. The exposure data were divided into different categories to show the exposure levels according to income areas and the product categories. Exposure was calculated for all the samples, milk source area, and respective income area for the consumer group to highlight the differences in exposure.

AFM_1_ levels in milk in the above studies were all analyzed with enzyme-linked immune-sorbent assay, using a commercial competitive ELISA (Helica AFM_1_ high sensitivity ELISA, Cat. No. 961AFLM01M-96) [27,36,45]. A total of 619 milk samples were analyzed for AFM_1_ levels.

For the risk assessment of stunting and cancer, distributions were fitted using @Risk 7.5 Industrial (Palisade Corporation, Ithaca, NY, USA) for the following categories: AFM_1_ levels in raw and processed milk and in total, in low-income areas, and in high-mid-income (mid-income) area; and milk consumption in total, in low-income areas, and in high-mid-income (mid-income) area, for adults and children, respectively.

Stochastic calculations were conducted using Monte Carlo simulations with 100,000 iterations in @Risk and the distributions for AFM_1_ levels and milk consumption best fitting to the reported consumption. When exponential distribution of milk consumption was used, the distributions were truncated to not exceed 3000 g for children and 4000 g for adults (Table S1 in supplementary materials lists all parameters). The body weight for adults was assumed to be 60 kg [59], and normally distributed with a 5 kg standard deviation, assuming a slight increase in average body weight [60]. Since milk consumption for children was collected for either below 2 or below 3 years of age, body weight was assumed to be 5–15 kg, and uniformly distributed. For the purpose of this study, we assumed that milk consumption and body weight were uncorrelated within age groups.

The cancer potency for aflatoxins has been assumed to be 0.01 cases per 100,000 people annually for each ng/kg bodyweight (bw) consumed per day, among people not infected with hepatitis B virus, and 30 times higher among those infected [14]. The prevalence of hepatitis-B-infected individuals used was 13% in Kenya based on earlier studies [14]. The risk for liver cancer was calculated for adults by multiplying the daily exposure with a worst-case and best-case potency and presented as the mean risk per 100,000 urban inhabitants and the overall Kenyan population with a 95% confidence limit.

Compared to AFB_1_, AFM_1_ is believed to be less carcinogenic, with at least 10 times less carcinogenicity [8], although both are classified as Group 1 carcinogens [2]. As the AFM_1_ data are limited, the AFB_1_ potency provides information estimation about the AFM_1_ potency. For this risk assessment, an estimate was done first using the estimate of potency suggested by the WHO, which is also an estimate 10 times lower, which then provides scenarios for cancer risks.

There are not many published associations between AFM_1_ in milk and stunting, but the estimate found by Kiarie et al. [27] in Kenya showed that the height-for-age adjusted z-score (HAZ score) decreased by 0.09 (standard deviation 0.045) for every increase of 1 ng AFM_1_/kg bw/day. This estimate is higher than found in other studies [49], but was used here for a worst-case scenario of the growth impact. The impact of AFM_1_ on HAZ score was assumed to be normally distributed but truncated at ±2 SD (thus only allowing the impact of AFM_1_ to vary between −0.18 and 0 for each increase in exposure) in order not to have extreme values for the sake of the model. The impact on HAZ for a child was calculated by multiplying the total exposure of AFM_1_ with this distribution, and then calculate the percentage that had a resulting HAZ of 2 or more out of the 100,000 iterations.

## Figures and Tables

**Table 1:** Reported milk consumption for adults in low-income (LI) and mid-income (MI) areas. Average consumption is calculated both for all the respondents and among those respondents who reported consuming milk.

Category	Number of Respondents N (%)	Daily Average mL (SD)	Annual Average (L)
All respondents	837 (100%)	437 (534)	160
Milk consumers	612 (73%)	589 (544)	214
LI respondents	543 (65%)	539 (599)	197
LI milk consumers	446 (82%)	657 (600)	240
MI respondents	294 (35%)	229 (294)	84
MI milk consumers	166 (56%)	406 (285)	148

**Table 2:** Milk consumption for children below three years old in low-income (LI) and mid-income (MI) areas. No children were reported to not consume milk at all.

Category	Number of Respondents N (%)	Daily Average mL (SD)	Annual Average (L)
All children	473 (100%)	438 (437)	160
LI children	391 (83%)	398 (451)	145
MI children	82 (17%)	626 (299)	229

**Table 3:** Aflatoxin M_1_ (AFM_1_) levels for milk samples from informal and formal dairy chains in low-income (LI) and mid-income (MI) areas, and samples exceeding the two most common limits of 0.5 ng/g and 0.05 ng/g.

Samples	N (%)	AFM_1_ (ng/g)			Samples above a Limit of	
		Mean	SD	Median	0.5 ng/g (%)	0.05 ng/g (%)
**All**	619 (100%)	0.105	0.195	0.059	19 (3%)	349 (56%)
**Raw milk ^1^**	368 (59%)	0.123	0.233	0.064	16 (4%)	225 (61%)
**Processed milk ^2^**	251 (41%)	0.079	0.116	0.049	3 (1%)	124 (49%)
UHT and pasteurized milk	178 (29%)	0.074	0.105	0.048	2 (12%)	86 (48%)
Fermented milk ^3^	73 (12%)	0.091	0.139	0.051	1 (1%)	38 (52%)
**LI milk**						
All LI milk	463 (70%)	0.119	0.215	0.064	18 (4%)	287 (62%)
LI processed milk	95 (15%)	0.102	0.127	0.064	2 (2%)	62 (65%)
LI raw milk ^1^	368 (59%)	0.123	0.233	0.064	16 (4%)	225 (61%)
**MI milk ^4^**						
Processed milk	156 (30%)	0.065	0.107	0.040	1 (1%)	62 (40%)

^1^ Raw milk samples were all from LI areas. ^2^ Processed milk includes samples from UHT (ultra-high temperature processed) milk, pasteurized and fermented milk products available in LI and MI areas. ^3^ Fermented milk includes samples from yoghurt and lala products. ^4^ Only processed milk samples were collected from MI area.

**Table 4:** Exposure to AFM_1_ through milk products from low-income (LI) and mid-income (MI) areas among adults.

Consumer	Milk Category	Exposure	
		ng/day	ng/kg bw/day
**All consumers**	**All milk**	46	0.8
**LI milk consumers**	**All milk**	69	1.2
	Raw milk	81	1.4
	Processed milk ^1^	52	0.9
	Pasteurized and UHT milk	49	0.8
	Fermented milk ^2^	60	1.0
	LI milk	78	1.3
	LI processed milk	67	1.1
**MI milk consumers**	**All milk**	43	0.7
	Processed milk ^1^	35	0.6
	Pasteurized and UHT milk	32	0.5
	Fermented milk ^2^	37	0.6
	MI milk	27	0.4

^1^ Processed milk includes samples from UHT, pasteurized and fermented milk products. ^2^ Fermented milk includes samples from yoghurt and lala products.

**Table 5:** Exposure to AFM_1_ through different milk products among children below three years old in low-income (LI) and mid-income (MI) areas. The exposure was calculated deterministically by multiplying mean contamination level with mean consumption level.

Consumer	Milk Category	Exposure	
		ng/day	ng/kg bw/day
**All children**	**All milk**	46	3.5
**LI children**	**All milk**	42	3.2
	Raw milk	49	3.8
	Processed milk ^1^	31	2.4
	Pasteurized and UHT milk	30	2.3
	Fermented milk ^2^	36	2.8
	LI milk	47	3.6
	LI processed milk	40	3.1
**MI children**	**All milk**	66	5.1
	Processed milk ^1^	50	3.8
	Pasteurized and UHT milk	47	3.6
	Fermented milk ^2^	57	4.4
	MI milk	41	3.2

^1^ Processed milk includes samples from UHT, pasteurized and fermented milk products. ^2^ Fermented milk includes samples from yoghurt and lala products.

**Table 6:** Annual risk for hepatocellular carcinoma (HCC) in per 100,000 people overall and then Kenyan population, assuming AFM_1_ carcinogenicity of 10 times less than AFB_1_, categorized between low-income (LI) and mid-income (MI) area consumers and milk category.

Cancer Risk	Per 100,000 (95% CI)	Kenya ^1^ (95% CI)
**All**	0.004 (0.000013–0.01)	1.7 (0.006–6.0)
**LI consumers**		
All milk categories	0.005 (0.000016–0.02)	2.0 (0.008–7.5)
LI milk	0.006 (0.000019–0.018)	2.7 (0.009–8.7)
**MI consumers**		
All milk categories	0.002 (0.000007–0.007)	0.9 (0.003–3.2)
MI milk	0.001 (0.000005–0.005)	0.6 (0.003–2.3)
Processed milk	0.003 (0.000012–0.011)	1.4 (0.005–5.3)
Raw milk	0.004 (0.000014–0.015)	2.0 (0.006–7.1)

^1^ Kenyan population is estimated 46,000,000 [35].

**Table 7:** Growth reduction as a reduction in mean height-for-age z-score (HAZ) in children related to AFM_1_ exposure from milk consumption categorized by low-income (LI) and mid-income (MI) areas.

Growth Reduction	HAZ (95% CI)	% Children −2 HAZ
**All children**	−0.340 (−1.254, −0.003)	2.7%
**LI children**		
All milk	−0.314 (−1.170, −0.003)	2.4%
LI milk	−0.358 (−1.297, −0.003)	2.8%
**MI children**		
All milk	−0.503 (−1.741, −0.014)	4.1%
MI milk	−0.337 (−1.136, −0.011)	2.1%

## Supplementary Materials

The following are available online at http://www.mdpi.com/2072-6651/10/9/348/s1, Table S1: The risk assessment parameters which were used in @Risk modeling.
