# Supplementary material for: A Risk Assessment of Aflatoxin M1 Exposure in Low and Mid-Income Dairy Consumers in Kenya
Source: Toxins (Basel). 2018 Aug 29;10(9):348. doi: 10.3390/toxins10090348 (PMC6162552; doi:10.3390/toxins10090348)
Supplement: Supplementary file 1 [file toxins-10-00348-s001.pdf]

Supplementary Materials: A risk assessment of aflatoxin M1 exposure in low and mid-income dairy consumers in Kenya

Sara Ahlberg, Delia Grace, Gideon Kiarie, Yumi Kirino, and Johanna Lindahl

Table S1. The risk assessment parameters which were used in @Risk modeling.

Consumer group		AFM_1 levels in milk	Milk consumption
**Adults**	**All**	RiskLogLogistic (0, 55.02, 1.6201)	RiskExpon (430.5) RiskTruncate (0, 4000)
	Mid-income	RiskLogLogistic (0, 39.532, 1.741)	RiskExpon (229.34) RiskTruncate (0, 4000)
	Low-income	RiskLogLogistic (0, 62.058, 1.6381)	RiskExpon (539.42) RiskTruncate (0, 4000)
**Children**	**All**	RiskLogLogistic (0, 55.02, 1.6201)	RiskExpon (438) RiskTruncate (0, 3000)
	Mid-income	RiskLogLogistic (0, 39.532, 1.741)	RiskTriang (0, 500, 1400)
	Low-income	RiskLogLogistic (0, 62.058, 1.6381)	RiskExpon (398) RiskTruncate (0, 3000)
